# Comparative Evaluation of Physical Surface Changes and Incidence of Separation in Rotary Nickel-Titanium Instruments: An *in Vitro* SEM Study

**Published:** 2014-07-05

**Authors:** Rudra Kaul, Riyaz Farooq, Vibhuti Kaul, Shafayat Ullah Khateeb, Aamir Rashid Purra, Roopali Mahajan

**Affiliations:** a Department of Conservative Dentistry and Endodontics, Institute of Dental Sciences, Jammu, India; b Department of Conservative Dentistry and Endodontics, Government Dental College and Hospital, Srinagar, India; c Department of Oral Medicine and Radiology, Government Dental College and Hospital, Srinagar, India

**Keywords:** Fatigue, Nickel-Titanium Alloy, Physical Surface Change, Profile, RaCe, Scanning Electron Microscopy, SEM, Stainless Steel, Torsional Force, Twisted File

## Abstract

**Introduction**: The aim of the present study was to comparatively evaluate the physical surface changes and incidence of separation in rotary nickel-titanium (NiTi) instruments using scanning electron microscope (SEM). **Methods and Materials:** A total number of 210 freshly extracted human maxillary and mandibular first molars were selected and distributed between three groups. Three different systems of rotary NiTi instruments, namely ProFile (PF), RaCe (RC) and Twisted File (TF), were used to prepare the canals using crown-down technique. All instruments were evaluated by means of SEM with 500× and 1500× magnifications, at four different stages; before use, after preparation of 7 and 14 canals and after instrument separation. Photomicrographs were also taken. The data was analyzed using the Kruskal-Wallis test and the level of significance was set at 0.001. It was found that H (HAT matrix) was 15.316 with 2 degrees of freedom. Moreover the various groups were compared using the Student-Newman-Keuls test with *P*<0.05 and it was found that all groups were significantly different. **Results:** RC showed the maximum wear of the surface followed by TF (*P*<0.05). PF showed the minimum wear except for its tip. There was no correlation between electropolishing and file fracture. Insignificant difference was observed in the mean number of canals shaped by PF and TF before their separation. **Conclusion:** Clinically, TF performance was superior, followed by PF then RC. RC fracture rate was the greatest after preparing the least number of canals.

## Introduction

Over the past fifteen years, a paradigm shift in root canal treatment has occurred with rotary nickel-titanium (NiTi) instruments becoming a part of the standard armamentarium of general practitioners and specialists [1]. In spite of their greater flexibility and more resistance to torsional forces than stainless steel (SS) instruments [2], separation of NiTi rotary instruments via torsional and cyclic fatigue is still possible and common, especially after extended use [3-5]. Unfortunately, many of these fractures happen unexpectedly without any visible signs of permanent deformation. Cyclic and static/dynamic torsional fatigue are the most common causes for fracture of rotary NiTi instruments [6].

The shape memory property and superelasticity of NiTi alloy made it impossible to manufacture files by twisting a NiTi wire in a specific molecular phase as it is done with SS K-files and K-reamers. Pioneer NiTi files such as ProFile (PF) (Dentsply Maillefer, Ballaigues, Switzerland), were traditionally manufactured by machining the original NiTi wire with carbide burs or silicon carbide wheels which can create imperfections such as grooves, pits, and metal fold-over on the cutting edges that serve as points of stress concentration and crack propagation [7, 8]. Manufactures of RaCe (RC) (FKG Dentaire, La-Chaux-de Fonds, Switzerland) claim that electropolishing after grinding and milling removes many of the machining defects on NiTi files and can inhibit stress concentration and crack propagation thereby can reduce file separation [9]. Twisted File (TF) (Sybron Dental Specialties, Orange, CA, USA) is manufactured with R-phase technology that represents advancement in the manufacturing of NiTi instruments where the imperfections due to the machining process are eliminated. The manufacturers claim that twisting of a R-phase NiTi wire increases its flexibility by 70% and reduces the chance of file separation [10]. Geometrical configuration of PF is U-shaped whereas both RC and TF have triangular cross-sections and this fact can affect their resistance to fracture.

**Table 1:** Preparation sequence for ProFile (WL=Working Length)

**Preparation step**	**Size **	**Preparation region **
**Coronal preparation**	40/0.06	Coronal third
	30/0.06	
**Crown-down sequence**	25/0.06	3 mm from the WL
	20/0.06	
	25/0.04	
	20/0.04	
**Apical preparation**	20/0.04	To WL
	25/0.04	

**Table 2:** Preparation sequence for RaCe (WL=Working Length)

**Preparation step**	**Size**	**Preparation region**
**Coronal** ** preparation**	40/0.10	Coronal third
	35/0.08	
**Crown-down sequence**	25/0.06	3 mm from the WL
**Apical preparation**	25/0.04	To WL

**Table 3:** Preparation sequence for Twisted File (WL=Working Length)

**Preparation step**	**Size**	**Preparation region**
**Coronal preparation**	25/0.08	Coronal third
**Crown-down sequence**	25/0.06	3 mm from the WL
**Apical preparation**	25/0.04	To WL

However, the claims of the manufacturers have not been adequately tested by independent researchers. The aim of this study was to evaluate the physical surface changes as well as the incidence of separation in three different systems of rotary NiTi instruments.

## Methods and Materials

This study was conducted at the Department of Conservative Dentistry and Endodontics, Government Dental College, Srinagar, India. Protocols of the Cross Infection Control (CIC) guidelines and the Occupational Safety and Health Association (OSHA) were observed at all stages. For this *in* *vitro* study, 105 freshly extracted maxillary first molars and 105 freshly extracted mandibular first molars were used. It was decided to use buccal canals of maxillary molars and mesial canals of mandibular molars which are small and tortuous enough for this study [11]. The teeth were selected according to the following criteria; first molars with intact coronal tooth structure and teeth indicated for extraction due to poor periodontal health. No age or gender consideration was included during selection of the teeth. Grossly carious or restored teeth and teeth with advanced attrition, abrasion, erosion, crown fracture, open apices, calcified canals and severe dilacerations, were excluded from the study. A total number of 420 canals were instrumented.

**Table 4:** Number of canals prepared before file fracture

**File number**	**RaCe**	**ProFile**	**Twisted File**
**1**	20	26	33
**2**	22	24	29
**3**	22	24	31
**4**	18	24	33
**5**	21	25	28
**6**	22	23	29

Also the angle and radii of curvature of canals was determined by a radiographic image obtained with Computed Dental Radiography (CDR) software for Microsoft Windows, Version 2.6 (Schick Technologies, Inc., NY, USA) and then transferred onto the AutoCAD software (Autodesk Inc., San Rafael, California, USA) based on an earlier described novel approach [12]. Canals having angle of curvature more than 30 degrees were excluded from the study.

***Distribution of samples in groups***

The teeth were randomly divided into 3 groups (*n*=60). Each set consisted of 30 maxillary first molars and 30 mandibular first molars. The groups were labeled as follows: group A; PF, group B; RC and group C; TF. The 60 teeth in each group were then divided into 6 subgroups *viz.* A1-6, B1-6 and C1-6. Each subgroup contained 10 teeth.

***Preparation of the canals***

The files were used in a crown-down technique using the electric motor (Endo-Mate TC, NSK, Nakanishi Inc., Tokyo, Japan) at a speed of 300 rpm with a clockwise rotation and a torque setting of 4.0 Nm. The preparation sequence for all the three file systems is given in Tables 1-4.

**Figure 1:**
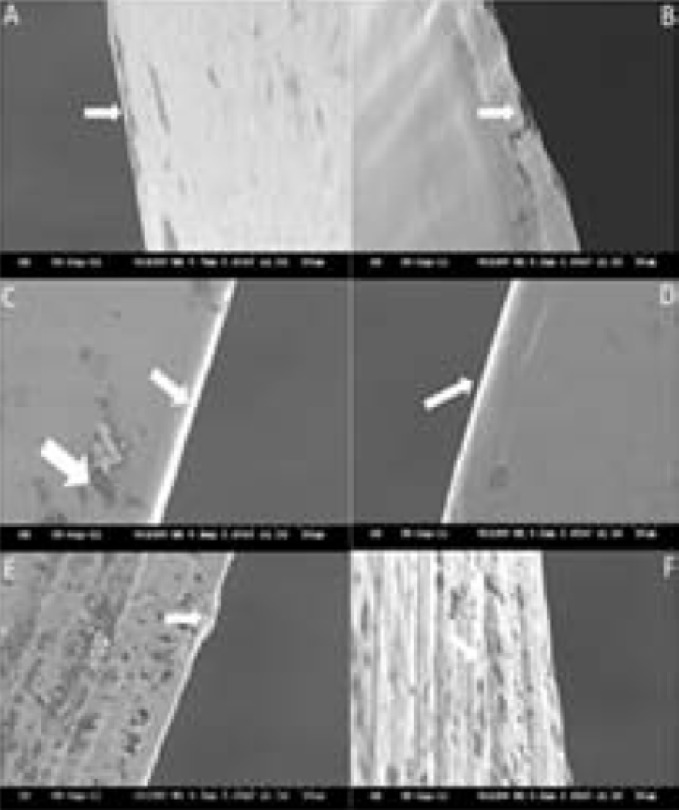
SEM of unused files; *A)* ProFile (PF); metal rollover; *B)* Mechanizing grooves on PF; *C *and *D)* Smooth electropolished surface of RaCe; *E)* Twisted File (TF) showing electropolished surface with defects; *F)* Unique surface texture of a TF resembling mechanizing grooves running through the file length

The master apical files of each group *i.e.,* PF 25/0.04; RC 25/0.04 and TF 25/0.04 were observed under the scanning electron microscope (SEM) (Hitachi, Ibaragi, Japan) for four times (*i.e.* before use, after preparation of 7 canals, after preparation of 14 canals and following instrument separation), to check the presence of deformation and machining defects, microcracks, metal rollovers, surface irregularities, *etc*. The total number of canals that 6 master apical files of a specific variety prepared before fracturing was calculated to see the extent of clinical performance. Separation within each group was recorded.

***Data analysis***

The data was analyzed using the Kruskal-Wallis test with the level of significance set at 0.001. Moreover the various groups were compared using the Student-Newman-Keuls test with *P*<0.05.

## Results

SEM observations of the surface characteristics and features of the new unused instruments did not reveal any microcracks on the cutting edge for all groups. PF showed certain machining marks along the faces of the flutes. RC showed less surface irregularities with remnants of the transverse running machining grooves vaguely discernible. Despite being electropolished by the manufacturer, the surface of TF was not perfectly smooth but showed a unique surface texture (Figure 1).

**Figure 2:**
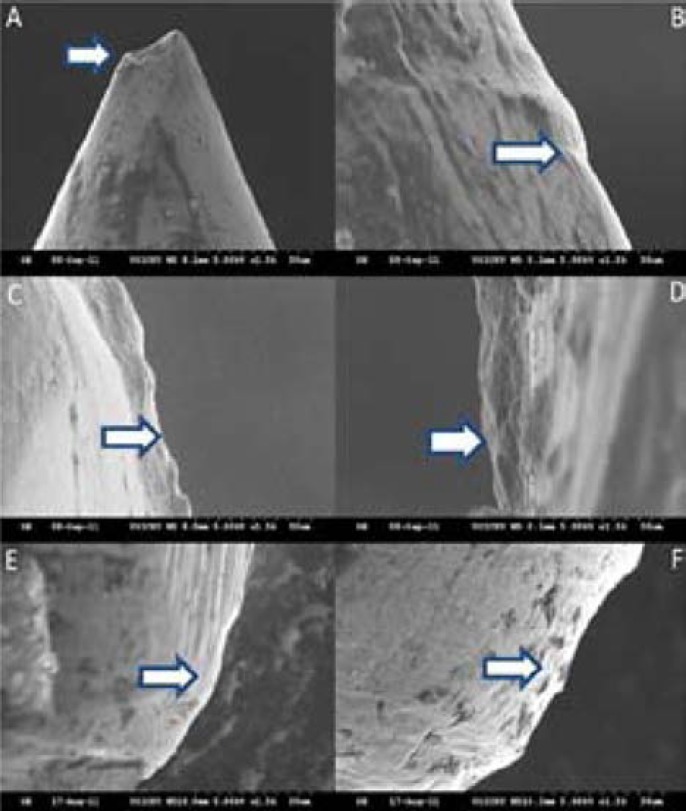
SEM of files after use in seven canals; *A)* Tip wear of ProFile (PF); *B)* Surface irregularities of PF; *C)* Nicks and notches on RaCe (RC); *D)* Highly abraded surface of RC; *E)* Abrasive marks on Twisted File (TF); *F)* Initiation of notches on TF

After instrumentation of seven canals, PF group exhibited excessive wear in the tip but the flute surfaces showed slight irregularities due to mild abrasion. RC files exhibited nicks and notches with an increase in the wear of flute edges. In the TF group small pitting and abrasive areas of greater magnitude were seen at the flute edge. Small metal shavings were also seen, thus rendering a rougher surface (Figure 2).

**Figure 3:**
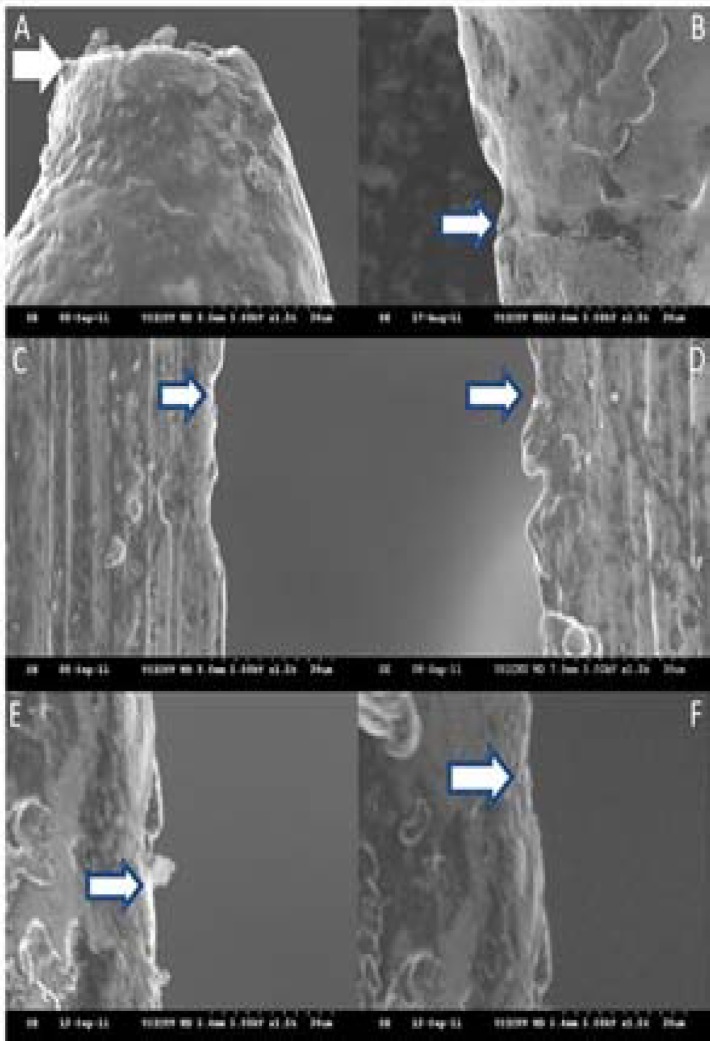
SEM image of files after preparing 14 canals; *A)* ProFile (PF) showing total tip loss; *B)* Crater on PF; *C)* Craters on RaCe; *D)* Microcracks with thin metal fins at the periphery of RC; *E)* Metal shavings on surface of Twisted File (TF); *F)* Microcracks and mechanizing grooves running throughout the length of the TF

After preparation of fourteen canals, PF showed wears in the forms of craters which neither left thin fins of metal at the periphery nor transmitted microcracks towards the body of the file. On the contrary the tips of the files showed excessive wear and was almost seen flat. RC group showed definite microfractures, which had propagated through the body of the file leaving the thin fins of metal at the surface of the flute. In the TF group the flute margins were irregular with further increase in the length and depth of abrasive areas that appeared as notches. Very frequently these notches were seen to facilitate microcracks into the body of the file (Figure 3).

**Figure 4:**
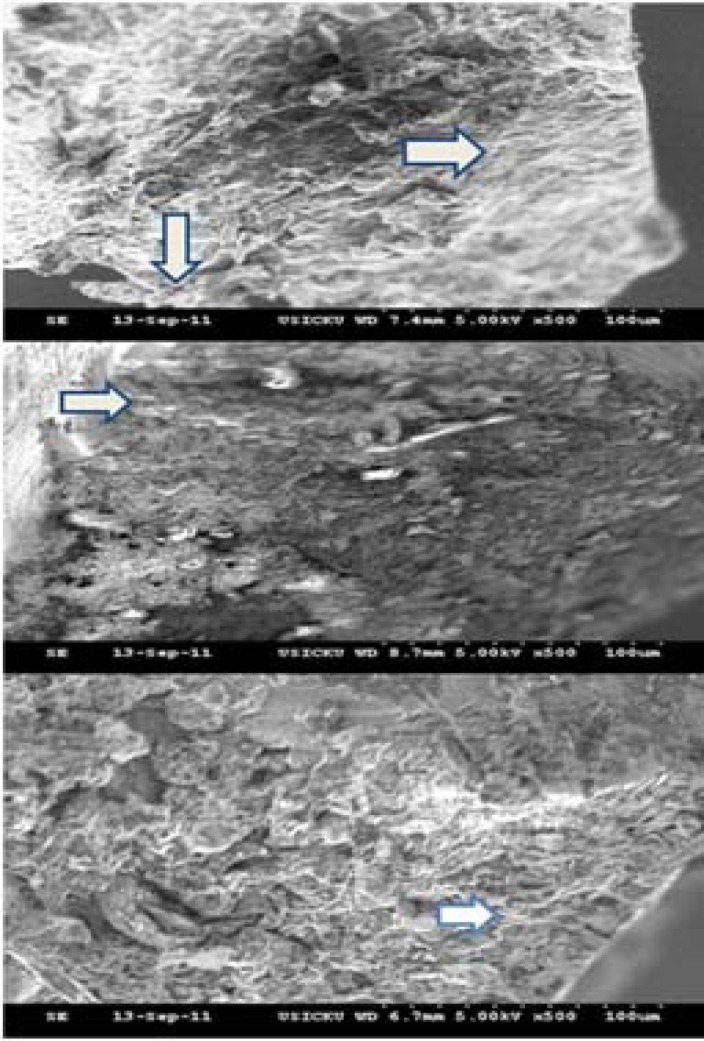
SEM of fractured surfaces of separated files; *A)* ProFile; loss of marginal integrity and concentric circles indicating shear fracture; *B)* RaCe; fatigue striations arising from the periphery of mechanizing grooves running throughout the file length; *C)* Twisted File; fatigue striations

Eighteen instruments from different groups fractured that were all examined under 500× magnification (Figure 4). Only 2 out of 18 files (11.1%) both belonging to PF group, showed torsional fracture and the rest showed fatigue fractures. In the center of the fracture surface irregular or skewed dimples were seen in areas that were not affected by fatigue striations or abrasion marks. Few specimens showed cracks that did not communicate with the periphery. This was considered as fatigue failure because of the presence of fatigue striations near the center of the cross section.

The results of this study clearly show that there are mechanizing grooves and other surface deformities present on non-electropolished NiTi files such as PF. Electropolished samples, *i.e.* RC, showed smooth and shiny surfaces with almost no surface deformity. TF showed a unique surface which resembled neither of the two. There were machining grooves throughout the length of the file.

RC showed the maximum wear/tear of the surface followed by TF. PF showed the minimum wear except for its tip. SEM analysis of the fractured surface of the separated files showed increased file fracture that was due to fatigue rather than shear. Inter-group comparison showed significant differences between the groups, with the maximum difference seen between PF and TF (*P*<0.05) (Tables 5 and 6).

The mean graph showed that TF performed better among the other three groups followed by PF and RC, respectively. RC fractured after preparing the least number of canals. After mean rank comparisons using the Kruskal-Wallis test, it was found that H (HAT matrix) was 15.316 with 2 degrees of freedom.

## Discussion

File fracture is the major concern during endodontic treatment. The structural characteristics and geometry have definite influence on the susceptibility of NiTi instrument to fracture [7, 13-16]. Keeping manufacturing factors aside, clinician’s handling is the main factor governing NiTi file fracture [17].

Besides the geometrical configuration, the importance of surface texture, such as mechanizing marks and scratches from manufacturing procedure has been implicated by a number of researchers [7, 17-20]. These mechanizing scratches on the instrument surface could act as local stress raisers or centers for formation of crack-like structures that can potentially become the origin of a fatigue crack; whereas smooth surfaces are less prone to initiation of fatigue fracture [7, 17, 20, 21]. The primary objective of the study was to analyze the files with qualitative and quantitative measures. While SEM evaluated files qualitatively, the number of canals prepared by each file before separation gave the quantitative insight into their clinical performance.

After analyzing the number of canals prepared before instrument separation, it was clear that there was no correlation between electropolishing and file fracture, as RC with highly electropolished surface shaped the least number of canals before separation. There was very low difference in the mean number of canals shaped before separation by PF and TF proving that the cross-section of the file and the method of manufacturing are more significant than the electropolished surface.

**Table 5:** Number of canals prepared before separation by different files

	**RaCe**	**ProFile**	**Twisted File**
**Mean**	20.83	24.33	30.5
**Standard Deviation**	1.60	1.03	2.16
**25** ^th^ ** Percentile**	20.25	24	29
**75** ^th^ ** Percentile**	22	24.75	32.5
**Median**	21.5	24	30

Applying this to the qualitative results obtained in the current study shows that under SEM, the unused non-electropolished instruments like PF, did not reveal any microcracks but metal rollovers and mechanizing grooves were seen. On the contrary, RC showed a smooth electropolished surface with fewer remnants of transverse running grooves and occasional pits [22, 23]. Despite DeOx surface treatment of TF (a proprietary process that removes the oxidation layer and any surface impurities but does not remove any of the base material) [24], the surface was not smooth but showed a unique surface texture resembling some kind of mechanizing groove. This can be explained by the manufacturer as a natural grain which is parallel to the long axis of the file and is preserved by twisting mechanism of the file-making procedure. As there are no mechanizing grooves and a DeOx layer is present which makes file resistant, the result is better clinical performance of the file.

After use in 7 and 14 canals there were fewer or no microcracks visible in PF. Unexpectedly both of the surface treated files showed lots of fretting, microcracks, nicking and notching and extensive crater formation. The extent and frequency of defects increased when the files were used in 7 canals and more. The fractured surface of all files showed quite similar surface features suggesting similar mechanism of fracture. In PF group there were frequent fracture defects and the cracks ended up in the triangular border [22]. Thus it can be assumed that the multitude of the mechanizing marks at multiple locations is greater than that required for crystallographic slip to occur. This observation is in agreement with the prevailing idea of propagation of cracks from the already established machining grooves in non-electropolished files, stated in other studies [7, 10, 18, 25]. On the other hand, the electropolished surface of RC removed all of the manufacturing defects but the resulting surface was weaker and more prone to newly initiated cracks. The DeOx coated surface of TF, however, was significantly more resistant to crack propagation.

Quantifying the clinical performance of all these files by counting the number of canals prepared before file separation, showed that TF had better performance than both RC and PF. This might be attributed to helical angel, the number of pitches and arrangement of spirals in the flute part [22]. That would describe such vast difference in the number of canals prepared by TF and RC in spite of having almost similar cross sections.

**Table 6:** Inter-group comparison (TF=Twisted File, PF=ProFile and RC=RaCe)

**Comparison**	**Difference of ranks**	**SE**	**P**	**q**	***P*** **-Value**
**TF ** ***vs.*** ** PF**	15.5-3.5=12	2.179	3	5.506	<0.05
**TF ** ***vs.*** ** RC**	15.5-9.5=6	1.472	2	4.076	
**RC ** ***vs. *** **PF**	9.5-3.5=6	1.472	2	4.076	

Moreover these phenomenal results of TF can also be due to its manufacturing process.

TF is manufactured by R-phase technology whereas RC and PF are made by grooving and milling. According to the manufacturer, TF instruments are made from a raw NiTi wire in the austenite crystalline structure and transforming it into rhombohedral R-phase by heat treatment process [23]. The transformation from austenite to R-phase is done by cooling the metal to the R-phase in transitional temperature; martensite begins to form on further cooling to and below the martensite start temperature. The rhombohedral structure with the material confined to a particular shape can be stabilized by applying a suitable holding temperature. R-phase shows good superelasticity (within about 1% strain) and shape memory. The Young’s modulus is typically lower than that of austenite. Thus an instrument made of R-phase would be tougher [26, 27]. The manufacturer claims that this R-phase technology represents advancement in manufacturing of the NiTi instruments. This twisting process with concurrent heat treatment, imparts superior mechanical characteristics than previous grinding and milling technique and this study provides back-up for this claim.

The current study is in accordance with the study by Herold *et al.* [21] stating that electropolishing did not prevent the formation of microcracks. The study even seconds the claim that Twisted File made by R-phase technology clinically perform better [22]. Anyway this study contradicts with several other studies that show electropolishing might improve the instruments working properties [25, 28]. The better performance of PF compared to RC, also suggests that instrument design has significant effects on its performance compared to its surface characteristics [29]. The recent years has seen a lot of contributions being made to the science of endodontology where SEM analysis has made it possible to evaluate changes at ultra-structural levels [30, 31]. Further advancements in this field will only be possible after unification of findings from such studies to recognize flaws in instrument engineering at the basic plane for optimized results.

## Conclusion

Electropolished files, such as RaCe, show a finished surface but wear off faster. ProFile initially shows machinizing grooves and metal rollovers but wears at a slower rate. Twisted File has unique surface characteristics and less wear than other files. Twisted File performed the best followed by ProFile and RaCe.
